# Effects of a structured nutrition education program on dialysis adequacy and clinical outcomes in maintenance hemodialysis patients

**DOI:** 10.3389/fnut.2026.1816453

**Published:** 2026-07-07

**Authors:** Desirée Victoria-Montesinos, Pura Ballester, Valentín Cadenas-García, Juana M. Morillas-Ruiz

**Affiliations:** 1 Universidad Catolica San Antonio de Murcia Facultad de Ciencias de la Salud, Murcia, Spain; 2 Servicio de Análisis Clínicos, Hospital Rafael Méndez de Lorca, Área de Salud III, Servicio Murciano de Salud, Murcia, Spain

**Keywords:** dialysis adequacy, Kt/v, maintenance hemodialysis, nutrition education, renal physiology

## Abstract

**Objective:**

To evaluate the effects of a structured nutrition education program on dialysis adequacy, dietary intake, metabolic parameters, and quality of life in patients undergoing maintenance hemodialysis, and to determine whether responses differ according to baseline dialysis adequacy.

**Methods:**

In this multicenter prospective study, patients receiving maintenance hemodialysis were allocated by center to either a nutrition education group, in which a researcher from the team delivered the site's standard nutrition education protocol, or to standard care without protocolized nutrition education, and were followed for 9 months. Participants were stratified according to baseline Kt/V (<25th percentile, 25th-75th percentile, and >75th percentile). Clinical, nutritional, biochemical, and functional outcomes were assessed at baseline and after the intervention period. Between-group comparisons were performed within each Kt/V stratum.

**Results:**

A total of 105 patients were included in the final analyses. Baseline characteristics were generally comparable between intervention and control groups across Kt/V strata. The response to nutritional education differed according to baseline dialysis adequacy. Patients with lower baseline Kt/V showed significant post-intervention improvements in dietary composition, including lipid quality and selected micronutrient intakes. In the intermediate Kt/V group, the intervention was associated with improvements in energy intake balance, systolic blood pressure, and selected hematological parameters. Patients with higher baseline Kt/V demonstrated significant post-intervention improvements in dialysis adequacy (Kt/V), body weight, hematological indices, physical function, and quality of life.

**Conclusion:**

A structured nutrition education program was associated with differential clinical and nutritional effects in patients undergoing maintenance hemodialysis, depending on baseline dialysis adequacy. These findings highlight the importance of considering baseline Kt/V when designing and evaluating nutritional education interventions in the hemodialysis setting.

## 1 Introduction

Chronic kidney disease (CKD) is a growing global health concern, affecting approximately 10% of the world's adult population (1, 2) and representing a major cause of morbidity and mortality (3, 4). CKD is etiologically heterogeneous, and in adults its most common causes include diabetes and hypertension, although many other metabolic, vascular, inflammatory, and structural disorders may also lead to progressive nephropathy (5). Among these causes, diabetic kidney disease is particularly relevant because it remains a leading contributor to CKD and end-stage kidney disease worldwide, with chronic hyperglycemia, oxidative stress, and microvascular injury involved in disease progression (4, 6). This is also consistent with the broader heterogeneity of diabetes phenotypes, including hereditary forms that increasingly require individualized clinical management (7).

In advanced stages, the irreversible decline of renal function requires renal replacement therapy, most commonly hemodialysis (HD). Although HD partially restores filtration capacity, it also imposes significant metabolic and nutritional challenges (8, 9). Patients undergoing maintenance HD frequently experience protein-energy wasting, micronutrient deficiencies, and chronic inflammation (10), all of which contribute to reduced survival and impaired quality of life (11). Malnutrition prevalence among HD patients is estimated to reach 40%−50% (12), and is attributed to multiple factors, including nutrient loss during dialysis, anorexia secondary to uremia, metabolic acidosis, comorbidities, and restrictive dietary prescriptions (13). These restrictions, particularly those limiting potassium, phosphorus, and protein intake, are essential for preventing metabolic complications (14), but often lead to poor dietary adherence and further nutritional decline.

Dialysis adequacy, commonly expressed as Kt/V, is a key clinical indicator of treatment effectiveness and a predictor of morbidity and mortality in HD patients (15). The parameter represents the fractional clearance of urea per session, integrating the dialyzer clearance rate (K), treatment time (t), and the volume of distribution of urea (V) (16). Achieving an optimal Kt/V is essential to prevent uremic toxicity, maintain metabolic homeostasis, and ensure patient survival. However, despite technological advances in dialysis equipment and treatment protocols, inadequate dialysis remains prevalent in many centers (16). Beyond technical parameters, individual behaviors such as dietary habits, fluid management, and adherence to medical recommendations substantially influence the efficiency of urea removal and, consequently, the Kt/V value.

Nutritional counseling is therefore considered a fundamental component of multidisciplinary renal care (17). The 2020 Kidney Disease Outcomes Quality Initiative (KDOQI) Clinical Practice Guideline for Nutrition in CKD emphasizes structured nutritional assessment, individualized medical nutrition therapy, and tailored management of protein, energy, electrolytes, and micronutrients in patients receiving maintenance dialysis (5). Recent observational evidence also shows that inadequate nutrient intake in maintenance HD is associated with poorer nutritional status and less favorable laboratory profiles (18). In line with this, a 2024 systematic review concluded that nutrition education interventions in HD patients can improve biochemical outcomes, dietary behavior, adherence, and quality of life, although intervention content and intensity vary substantially across studies (19). A more recent systematic review and meta-analysis likewise found that patient education in HD improves adherence to dietary and fluid restrictions and may enhance quality of life (20). Importantly, a recent randomized clinical trial reported that individualized patient education improved both HD adequacy (Kt/V) and interdialytic weight gain, supporting the plausibility that education may influence dialysis-related outcomes in addition to dietary behavior (21). Recent qualitative work further suggests that dietary adherence in HD depends not only on knowledge of potassium, phosphate, and protein sources, but also on thirst management, food preferences, and access to renal dietitian support (22).

Patient education may help individuals to better understand how dietary choices influence metabolic balance and dialysis-related outcomes, thereby improving adherence to dietary and therapeutic recommendations (19, 20). Several studies have shown that structured nutrition education programs (NEP) improve biochemical parameters, dietary behavior, and quality of life among patients receiving HD (19, 20). Educational strategies that incorporate individualized counseling, practical workshops, and behavioral reinforcement appear particularly relevant in this setting, where patients frequently face barriers related to food restrictions, thirst management, and limited access to renal dietitian support (22, 23). Mediterranean-style dietary principles have also been discussed in CKD as a potentially useful framework for improving dietary quality when adapted to the metabolic constraints of kidney disease (24–26). However, although nutritional education interventions have been shown to influence biochemical and nutritional parameters in HD patients (18, 27), the specific impact of such programs on dialysis adequacy, commonly measured by Kt/V, has not been adequately investigated (19).

The physiological mechanisms linking nutritional behavior and dialysis efficiency are multifactorial (28). Dietary protein intake directly determines urea generation, while hydration status and inter-dialytic weight gain affect urea distribution and clearance (29). Poor adherence to dietary recommendations can alter these factors, leading to fluctuations in solute removal efficiency. Conversely, optimized dietary patterns and improved self-management may enhance metabolic control and favor more effective urea clearance. In this context, integrating a NEP into standard dialysis care could not only mitigate biochemical disturbances but also improve dialysis performance as reflected by Kt/V.

Despite consistent evidence supporting the role of dietitians and nutritional counseling in CKD management (5, 30), interventional studies specifically evaluating whether structured educational approaches can modify dialysis adequacy across patients with different baseline Kt/V values remain scarce (5, 21). This question is clinically relevant because many patients on HD face persistent barriers to dietary adherence, including restrictive food rules, thirst management, food preferences, and insufficient access to ongoing renal dietitian support (22, 23). Thus, patient-centered educational interventions that combine practical guidance with ongoing reinforcement are needed to promote sustainable behavioral changes and to determine whether these changes translate into better dialysis-related outcomes.

The present study aimed to assess the effect of a structured NEP on dialysis adequacy, expressed as Kt/V, in patients undergoing maintenance HD. Secondary objectives included evaluating changes in biochemical, anthropometric, and pharmacologic parameters following the intervention. We hypothesized that the educational program would enhance patients' understanding of dietary management, promote better metabolic control, and ultimately result in improved dialysis efficacy.

## 2 Materials and methods

### 2.1 Study design and participants

This multicenter, prospective, and educational intervention study, in which a researcher from the team delivered the site's standard nutrition education protocol in the nutrition education group, was conducted in southeastern Spain over a 9-month period in three HD Centers: the Hemodialysis Unit of Lorca, the Hemodialysis Unit of San Juan de Alicante, and the Nephrology Department of Hospital Rafael Méndez. The main objective was to evaluate the effect of a structured NEP on dialysis adequacy, expressed as Kt/V, in patients undergoing maintenance HD. Secondary outcomes included biochemical, anthropometric, and pharmacologic parameters related to nutritional status and metabolic control. Data collection was carried out at baseline and after three, six, and 9 months of follow-up. Participants were allocated by dialysis center rather than by individual randomization in order to reduce contamination between patients treated within the same unit during the educational intervention.

A total of 105 adult patients were included. All participants had been receiving maintenance HD for at least 6 months and were clinically stable at the time of recruitment. Exclusion criteria included active infection, malignancy, severe cognitive impairment, psychiatric illness, hospitalization within the previous 3 months, or participation in another study. Patients were fully informed about the purpose and procedures of the study and voluntarily provided written consent to participate.

### 2.2 Nutrition education program

Participants were assigned to one of two groups according to their treatment center. The control group, consisting of 53 patients from the Hospital Rafael Méndez and San Juan de Alicante centers, continued receiving routine nephrology follow-up and the usual non-structured dietary recommendations customarily provided during standard clinical care. They did not receive protocolized educational workshops, study-specific written educational materials, or scheduled reinforcement sessions. The intervention group, composed of 52 patients from the Lorca unit, participated in a structured NEP coordinated by a multidisciplinary team including a nephrologist, a registered dietitian, and specialized renal nursing staff.

The NEP aimed to enhance patients' nutritional knowledge, self-management skills, and adherence to dietary recommendations through a combination of theoretical instruction and practical sessions. During the 1 month, participants attended three workshops covering key aspects of renal nutrition, adequate energy and protein intake, management of phosphorus, potassium, and sodium, and strategies for fluid balance. The sessions also emphasized portion size, meal planning, and cooking techniques to reduce mineral content (e.g., soaking or double boiling of vegetables). Patients were encouraged to prioritize fresh foods, limit processed products, and adapt their meal plans to individual preferences and cultural habits. Educational materials such as printed booklets, recipes, and food exchange tables were distributed.

Each workshop combined group lectures with interactive case discussions and hands-on demonstrations (e.g., cooking methods, label reading). During the intervention period, participants received individualized follow-up sessions. According to the original intervention protocol from the site, the nutritionist visited patients every 4–6 weeks during the first 3 months and monthly from month 4 to month 9. During these visits, the dietitian reviewed dietary recalls, reinforced behavioral strategies, and adjusted recommendations based on biochemical results and adherence. The intervention followed Mediterranean diet principles, fluid and sodium management, and coping strategies for common dietary challenges in HD, those already defined by the center. Educational activities were delivered during the HD sessions and were supported by standardized didactic materials, including PowerPoint presentations, images, and videos adapted to the patients' educational level. Informative posters were displayed in the dialysis and waiting rooms, and written take-home material summarizing the recommendations was provided to patients and relatives for home review. In addition to the group sessions, personalized nutritional counseling was offered whenever patients required further clarification or adaptation of recommendations. Family members were invited to selected sessions to foster a supportive environment, and the responsible physician and renal nursing staff were also present during the educational sessions. Although the intervention schedule and educational materials were standardized, the exact duration of each educational session and individual attendance/adherence rates were not prospectively recorded.

To avoid potential confounding, dialysis prescriptions (including session duration, dialyzer type, and blood flow rate) were maintained unchanged for all participants during the study period.

### 2.3 Outcome measures

The primary outcome was dialysis adequacy, assessed as single-pool Kt/V using the Daugirdas equation and calculated from pre- and post-dialysis serum urea concentrations obtained during routine laboratory testing. Blood samples were collected monthly under fasting conditions before and immediately after the mid-week dialysis session.

Secondary outcomes included biochemical, anthropometric, pharmacologic, and self-reported parameters. Serum concentrations of potassium, phosphorus, calcium, parathyroid hormone, albumin, hemoglobin, urea, and creatinine were analyzed in hospital laboratories using a Beckman Coulter AU5800 autoanalyzer (Beckman Diagnostics, Brea, CA, USA). Internal and external quality controls were performed to ensure analytical reliability. For hematological determinations, samples were processed in tubes containing ethylenediaminetetraacetic acid (EDTA) and analyzed using a Sysmex XE-5000 (Roche Diagnostics, Germany), with hemoglobin determined by the sodium lauryl sulfate (SLS) method. Blood samples were centrifuged in a Jouan C4i Thermo Scientific^®^ centrifuge at 3,500 g for 10 min prior to analysis.

Anthropometric parameters [body weight, height, and body mass index (BMI)] were measured using a digital scale with integrated stadiometer (Seca, Hamburg, Germany), with patients wearing light clothing and no shoes. All measurements were taken by trained personnel following the International Society for the Advancement of Kinanthropometry (ISAK) and Spanish Group of Cineanthropometry recommendations. Inter-dialytic weight gain was calculated as the difference between the post-dialysis weight of the previous session and the pre-dialysis weight of the following session. Dietary intake was assessed at baseline and during follow-up using 24-hour dietary recalls conducted by the dietitian, and energy and nutrient composition were analyzed with Dietsource^®^ software (Novartis, Spain). At each dietary assessment point, six 24-h dietary recalls were collected per participant: four on non-dialysis days, including one holiday day, and two on dialysis days. Because food intake on dialysis days was frequently altered when treatment schedules overlapped with usual meal times, resulting in food choices that were not representative of the participants' habitual dietary intake, recalls collected on dialysis days were excluded from the nutritional analysis. Therefore, energy and nutrient intake were estimated as the arithmetic mean of the four non-dialysis recalls. Portion sizes were estimated using standardized food photographs with predefined food weights and conventional household measures commonly used in the local setting. All recalls were administered face to face by the same nutritionist following the same procedure, which helped minimize inter-interviewer variability in portion size estimation and dietary data collection.

Information about prescribed medication—including phosphate and potassium binders, vitamin D analogs, and erythropoiesis-stimulating agents—was extracted from medical records. In addition, patients completed the Nottingham Health Profile (NHP) to evaluate perceived health status and the short version of the International Physical Activity Questionnaire (IPAQ) to assess habitual physical activity.

### 2.4 Clinical and laboratory procedures

All participants followed their regular HD schedule of three sessions per week, each lasting approximately 4 hours. Dialysis was performed using Fresenius 4008B^®^ monitors and high-flux synthetic dialyzers (Nipro^®^ ELISIO 170 or 190 DH polysulfone membranes, single-use), selected according to patient body weight (< 65 kg: ELISIO 170 DH; ≥65 kg: ELISIO 190 DH). The dialysate composition was standardized across all centers (bicarbonate 35 mmol/L, calcium 1.5 mmol/L, potassium 1.5 mmol/L, sodium 140 mmol/L, glucose 1 g/L, osmolarity 295 mmol/L). Blood flow ranged from 350 to 500 mL/min, and dialysate flow from 500 to 800 ml/min. Vascular access was a native arteriovenous fistula in all patients. Anticoagulation was achieved using bemiparin sodium (Hepadren^®^) administered as a single bolus at the start of each session, with dosage adjusted to body weight.

Blood pressure was measured before and after each dialysis session using an automated sphygmomanometer under standardized conditions. All biochemical and hematological analyses were conducted in certified hospital laboratories with quality assurance programs.

All laboratory, anthropometric, and dietary data were entered into a dedicated database and verified for completeness before statistical analysis.

### 2.5 Statistical analysis

For dietary variables, the arithmetic mean of the four non-dialysis 24-h recalls collected at each assessment point was calculated for each participant and used as the representative intake value in the statistical analyses.

An *a priori* sample size calculation was performed for the overall comparison between the nutrition education and standard care groups, considering Kt/V as the primary outcome. The study was therefore designed to detect between-group differences in Kt/V at the overall study-population level. However, the Kt/V-stratified subgroup analyses were exploratory and were not specifically powered; therefore, these subgroup findings should be interpreted with caution.

To evaluate the differential effect of the intervention according to baseline dialysis adequacy, participants were stratified into subgroups based on their initial Kt/V values. For the intervention group, the complete-case sample available for the Kt/V-stratified post-intervention comparisons was distributed as follows: Q1 (low, 0–25% Kt/V: 0–1.4, *n* = 10 participants), Q2 (intermediate, 26–75% Kt/V: 1.5–2.4, *n* = 26 participants), and Q3 (>75% high Kt/V: ≥2.5, *n* = 17 participants). These strata indicate progressively higher baseline dialysis adequacy, with Q1 representing lower dialysis adequacy, Q2 an intermediate status, and Q3 higher baseline dialysis adequacy. These cut-offs correspond to quartile thresholds; for analytical purposes, the two middle quartiles (P25–P75) were combined into a single intermediate group (Q2). This stratification was performed *a priori* to identify subgroups with potentially different capacities for response to the NEP. Data were collected at baseline and at 3, 6, and 9 months. The analyses were performed separately at each assessment time point within each baseline Kt/V stratum. Between-group comparisons were conducted at each time point using one-way analysis of variance (ANOVA) for normally distributed variables and Kruskal-Wallis tests for non-normally distributed variables. In addition, within-group changes across follow-up were explored through pairwise comparisons between assessment points. No formal repeated-measures ANOVA or mixed-effects model was applied. Statistical significance was set at *p* < 0.05. All analyses were performed using IBM SPSS Statistics, version 29.0 (IBM Corp.).

### 2.6 Ethical considerations

The study was conducted in accordance with the ethical principles of the Declaration of Helsinki. The research protocol was approved by the Research Ethics Committee of Universidad Católica de Murcia with reference code CE1872014. All participants were fully informed about the study objectives and procedures and voluntarily provided written informed consent for participation and for the use of their anonymized clinical data for research purposes. The structured NEP was implemented as part of standard clinical practice and aimed to enhance patients' understanding and adherence to dietary recommendations. The intervention did not involve any experimental procedures or deviations from routine care, and all participant data were handled confidentially and in accordance with applicable data protection regulations.

## 3 Results

**Figure 1:**
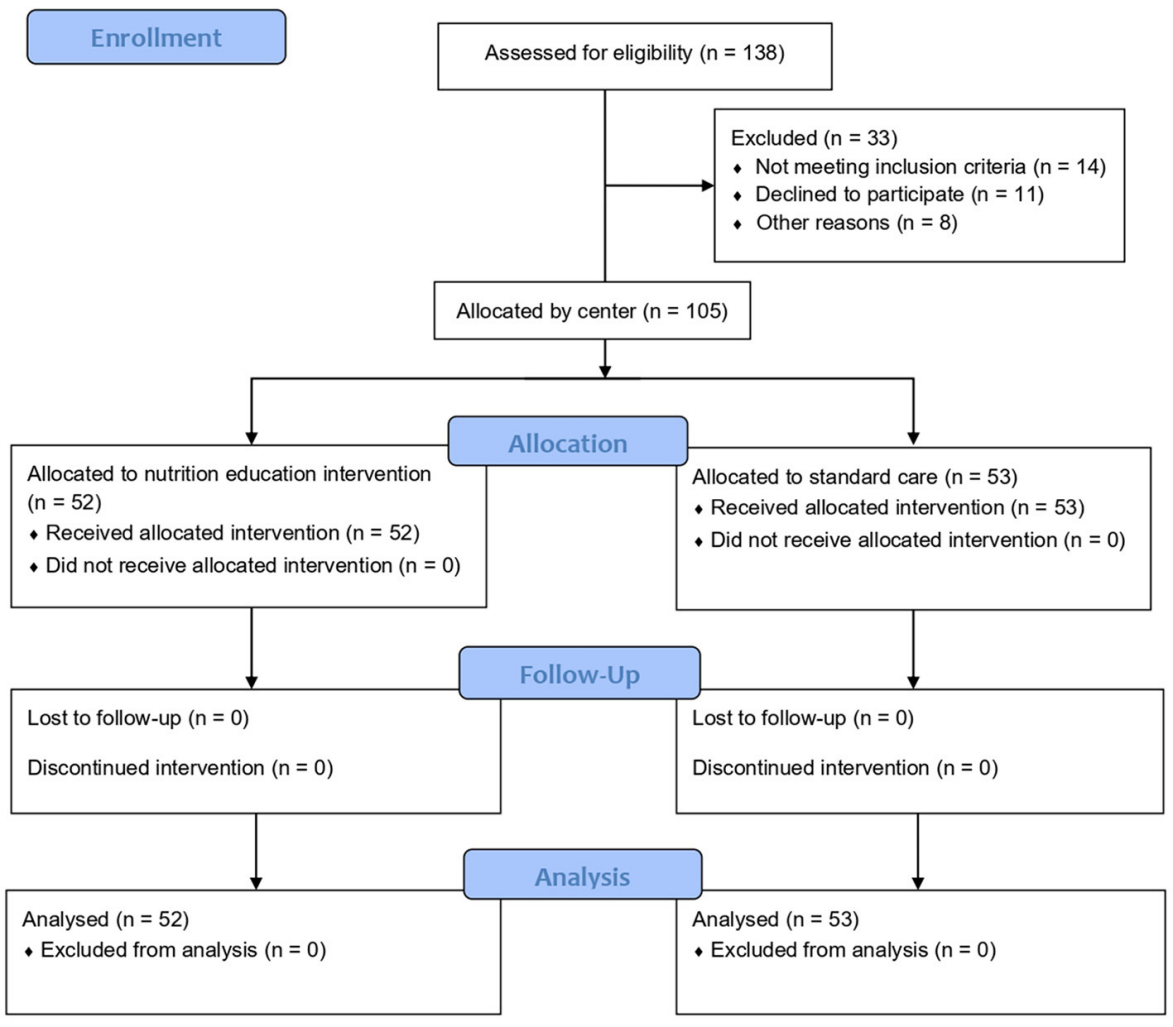
Flow diagram of participant recruitment, allocation, follow-up, and analysis. A total of 138 patients were assessed for eligibility, 33 were excluded, and 105 patients were allocated by center to the nutrition education intervention group (*n* = 52) or the standard care/control group (*n* = 53). All participants completed follow-up and were included in the final analysis.

A total of 138 patients were assessed for eligibility; 33 were excluded due to not meeting inclusion criteria (*n* = 14), declining participation (*n* = 11), or other reasons (*n* = 8). A total of 105 patients were included and allocated by center to the nutrition education intervention group (*n* = 52) or the control group (*n* = 53). All participants completed follow-up and were included in the final analyses (Figure 1, Table 1).

**Table 1:** Demographic characteristics of study sample of participants.

Characteristic	Intervention (*n* = 53)		Control (*n* = 52)	
	M	SD	M	SD
Age	67, 0	14, 3	62, 3	14, 5
MNA	21, 2	2, 8	21, 3	2, 6
SBP basal	127, 2	14, 4	125, 7	9, 2
DBP basal	82, 5	7, 0	81, 0	5, 9
Nottingham	45, 8	23, 8	45, 8	22, 3
IMC	21, 04	2, 19	22, 08	3, 47
Weight basal	70, 4	8, 7	71, 8	12, 5

The study population consisted of patients undergoing maintenance HD who were stratified into three groups according to baseline dialysis adequacy, defined by Kt/V values < P25, P25-P75, and >P75. Within each stratum, participants were assigned to either the intervention or control group. Baseline comparability between intervention and control groups was generally preserved across Kt/V strata. An exception was observed in the intermediate Kt/V group, in which baseline mean corpuscular volume differed significantly between groups (Mann-Whitney U test, *p* = 0.001).

Across all groups, mean age ranged from 61.1 to 69.1 years. Nutritional status, assessed using the Mini Nutritional Assessment, was comparable between groups and indicated preserved nutritional status (mean range: 20.9–21.8). Baseline physical function, measured using the Nottingham scale, showed variability within groups (mean values ranging from 39.1 to 55.4, SD = 14.5–27.7), without a consistent pattern favoring either intervention or control groups. Baseline systolic blood pressure ranged from 122.4 to 136.0 mmHg, while baseline diastolic blood pressure ranged from 79.4 to 83.0 mmHg, with no relevant differences between groups.

Following the intervention period, systolic and diastolic blood pressure values remained stable in both intervention and control groups. Final Kt/V values ranged from 1.6 to 3.0, consistent with the predefined stratification by dialysis adequacy. Parathyroid hormone concentrations decreased from baseline values (159.6–249.8 pg/ml) to post-intervention values (112.6–176.3 pg/ml) across most groups. Changes in phosphate binder use between baseline and post-intervention were limited (variation range: −0.7 to 0.4), whereas potassium binder use showed a more marked reduction in the intervention groups compared with controls.

Baseline energy requirements were similar across groups, ranging from 2,434.7 to 2,573.9 kcal/day. Actual energy intake at baseline was substantially lower than estimated requirements in all groups. In the intervention group, baseline energy deficits ranged from −919.9 to −1,038.5 kcal/day. After the intervention, energy intake increased modestly, with persistent deficits ranging from −691.5 to −1,161.1 kcal/day. A significant reduction in energy deficit was observed in the intermediate Kt/V group. Vitamin C intake decreased slightly from baseline (57.2–97.9 mg) to post-intervention values (57.3–87.4 mg) across groups. Intake of B vitamins (thiamine, vitamin B6, and vitamin B12) remained largely stable between baseline and post-intervention assessments, with the exception of the highest Kt/V group. Baseline potassium intake ranged from 1,433.0 to 3,326.8 mg/day and showed modest reductions following the intervention. Baseline hemoglobin concentrations ranged from 11.1 to 12.0 g/dL and remained stable post-intervention (11.0–12.3 g/dL). Mean corpuscular volume did not differ significantly between groups at baseline or post-intervention.

### 3.1 Post-intervention outcomes in Q1 group

In the Q1 group (first Kt/V group), several post-intervention differences were observed between intervention and control groups (Table 2). Phosphate binder use differed significantly (*p* = 0.008). Significant differences were also observed in total lipid intake (*p* = 0.011), saturated fat intake (*p* = 0.015), the difference between ingested and required lipid intake (*p* = 0.011), monounsaturated fat intake (*p* = 0.001), and polyunsaturated fat intake (*p* = 0.009). Post-intervention potassium intake differed significantly between groups (*p* = 0.013), as did vitamin C intake (*p* = 0.008).

**Table 2:** First Quartile post-intervention comparisons.

Outcome	Intervention		Control		*p*-value
	M	SD	M	SD	
% saturated fat intake post	6, 2	1, 8	9, 0	3, 3	0, 015
Diff % lipids (intake-required) post	−4, 2	5, 3	2, 6	7, 7	0, 011
% MUFA intake post	9, 3	2, 6	16, 5	6, 2	0, 001
% PUFA intake post	2, 1	0, 7	2, 8	0, 7	0, 009
Potassium intake post	2,218, 8	704, 4	1,501, 6	597, 9	0, 013
Vitamin C intake post	87, 4	15, 5	57, 3	29, 7	0, 008

### 3.2 Post-intervention outcomes in Q2 group

In the second Kt/V group, post-intervention systolic blood pressure differed significantly between intervention and control groups (*p* = 0.032) (Table 3). Differences were also observed in phosphate binder use (*p* = 0.010) and mean corpuscular volume (*p* = 0.002). Polyunsaturated fat intake differed significantly between groups (*p* = 0.038). Thiamine intake was higher in the intervention group (*p* = 0.049). Three-day post-intervention energy intake showed a significant between-group difference (*p* = 0.004), as did the balance between ingested and required energy intake (*p* = 0.018). Potassium intake showed a borderline difference between groups ((*p* = 0.057).

**Table 3:** Second quartile post-intervention comparisons.

Outcome	Intervention		Control		*p-*value
	M	SD	M	SD	
SBP 3	121, 9	11, 4	127, 4	9, 9	0, 032
MCV 3	104, 2	5, 2	99, 8	5, 0	0, 002
Energy (intake-required) post	−691, 5	903, 3	−1134, 7	409, 4	0, 004
% saturated fat intake pre	14, 6	25, 0	13, 0	20, 4	0, 018
g PUFA intake post	5, 7	2, 4	4, 3	1, 9	0, 038
Potassium intake post	1877, 6	450, 0	1647, 0	439, 9	0, 057
Thiamine B1 intake post	0, 7	0, 2	0, 8	0, 2	0, 049

### 3.3 Post-intervention outcomes in Q3 group

In the third Kt/V group, post-intervention differences between intervention and control groups were observed for Nottingham physical function score (*p* = 0.014), body weight (*p* = 0.001), and Kt/V (*p* = 0.010) (Table 4). Hemoglobin concentrations differed significantly between groups (*p* = 0.031), as did mean corpuscular volume (*p* < 0.001). Significant differences were also observed for thiamine intake (*p* = 0.002) and vitamin B6 intake (*p* = 0.017), while vitamin B12 intake showed a marginally significant difference (*p* = 0.047).

**Table 4:** Third quartile post-intervention comparisons.

Outcome	Intervention		Control		*p*-value
	M	SD	M	SD	
NOTTINGHAM	39, 1	14, 5	55, 4	14, 7	0.014
Weight index 3	2, 1	0, 6	2, 7	0, 3	0.001
Kt/V 3	3, 0	1, 3	2, 0	0, 4	0.010
Hemoglobin 3	12, 3	0, 7	11, 3	1, 3	0.031
MCV 3	103, 2	3, 8	95, 1	5, 2	< 0.001
Thiamine B1 intake post	0, 6	0, 1	0, 9	0, 2	0.002
Vitamin B6 intake post	0, 6	0, 1	0, 8	0, 3	0.017
Vitamin B12 intake post	1, 9	0, 4	2, 1	2, 3	0.047

## 4 Discussion

A clinically relevant finding of this study is that improvements in dialysis adequacy, as reflected by Kt/V at 3 months, were more evident among patients with higher baseline adequacy values (Q3; Kt/V ≥2.5). This observation contrasts with the common assumption that nutritional education mainly benefits patients with poorer baseline dialysis parameters and suggests a non-linear interaction between dietary interventions and baseline physiological conditions (31–33). While previous studies have primarily focused on quality of life, adherence, and biochemical outcomes (27, 34), evidence linking nutrition education to changes in dialysis adequacy remains limited. In this context, our findings are consistent with those of Sajjadi et al. (21), who reported improvements in both interdialytic weight gain and Kt/V following individualized educational interventions.

The heterogeneous response across baseline Kt/V groups suggests that nutritional education may act through different physiological pathways depending on dialysis adequacy. The pattern observed in Q3 points to a possible effect on dialysis-related behaviors rather than on dialysis prescription itself.

In patients with higher baseline Kt/V (Q3), the observed increase in Kt/V may be related to volume-associated mechanisms. Because Kt/V is determined by solute clearance relative to urea distribution volume (V), reductions in V in the absence of changes in dialysis prescription may translate into higher achieved Kt/V (15, 35). In this study, Q3 patients showed concurrent increases in Kt/V and reductions in body weight index. This pattern may be compatible with improved fluid management rather than changes in lean body mass. The HEMO Study identified modeled urea distribution volume as an independent predictor of mortality (35). However, because body composition and objective volume status were not directly assessed, this interpretation remains speculative and should be considered hypothesis-generating.

Improved sodium awareness and fluid management may have contributed to lower interdialytic fluid accumulation and more stable ultrafiltration, factors associated with improved tolerance to prescribed treatment time and effective solute clearance (36–40). However, intradialytic complications, body composition, and objective fluid status were not directly assessed in the present study. Therefore, this interpretation should be considered cautious and hypothesis-generating rather than confirmatory (41).

In contrast, patients with the lowest baseline Kt/V (Q1) did not show significant changes in dialysis adequacy or hemodynamic parameters. However, they did show meaningful improvements in dietary composition. Reductions in saturated fat intake and increases in monounsaturated and polyunsaturated fatty acids indicate improved dietary decision-making and adherence to Mediterranean-style dietary recommendations adapted for HD (26, 42–45). These qualitative changes are unlikely to modify dialysis adequacy in the short term. Nevertheless, they may still be clinically relevant because of their potential effects on inflammation, endothelial function, and vascular stability (46–48). The higher vitamin C intake probably reflects improved food selection rather than dietary liberalization, given potassium restrictions in this population.

Patients in the intermediate baseline Kt/V group (Q2) showed a broader response pattern. The intervention was associated with metabolic, hemodynamic, and hematological changes, suggesting that this subgroup may represent a physiological window in which multiple pathways remain modifiable. Reductions in systolic blood pressure are consistent with improved sodium and volume management, a key determinant of blood pressure control in HD patients (49). This may also contribute to better intradialytic stability and treatment efficiency (50). In parallel, reductions in energy deficit may have mitigated maladaptive protein catabolism, a central feature of protein-energy wasting (10, 51, 52). This interpretation is supported by increases in mean corpuscular volume and improved intake of thiamine and other B vitamins, which are essential for erythrocyte maturation (53–57). The concomitant increase in polyunsaturated fatty acid intake further suggests an overall improvement in dietary quality.

Hematological improvements were most evident in Q2 and Q3. In these groups, increases in hemoglobin and mean corpuscular volume coincided with higher intakes of thiamine, vitamin B6, and vitamin B12. Adequate intake of these micronutrients is critical for effective erythropoiesis, even in patients receiving erythropoiesis-stimulating agents (53, 54, 56). Improved energy balance may have potentiated these effects by reducing excessive protein catabolism and urea generation (29, 51). However, these mechanisms should be interpreted as biologically plausible rather than definitively demonstrated.

Phosphorus management also appeared to respond in a baseline Kt/V-dependent manner. Reductions in phosphate binder use in Q1 and Q2 are consistent with improved understanding of dietary phosphorus sources and greater adherence to avoidance-based strategies, particularly limiting ultraprocessed foods containing phosphate additives (14, 58–60). Importantly, these improvements were not accompanied by deterioration in nutritional status, suggesting that education can help reconcile phosphorus restriction with adequate protein intake (58, 59).

Finally, improvements in quality of life were confined to patients in Q3. In this subgroup, several objective benefits converged, including improved Kt/V, lower body weight index, enhanced hematological parameters, and optimized micronutrient intake. Given the multifactorial nature of quality of life in HD patients (61–64), individuals with higher baseline adequacy may have sufficient physiological reserve to perceive incremental improvements. By contrast, patients with lower adequacy may require more pronounced or sustained changes before such benefits become evident. Increased self-efficacy and empowerment through nutritional knowledge acquisition may also contribute to these effects (22, 65).

Overall, these findings indicate that the effects of nutritional education in HD patients are context-dependent and shaped by baseline dialysis adequacy. The intervention did not produce the same type of response in all patients. Instead, the dominant effect appeared to vary according to baseline status. In patients with lower adequacy, the main response was an improvement in dietary quality. In intermediate patients, the intervention was associated with broader metabolic and hemodynamic changes. In patients with higher adequacy, the clearest signal involved dialysis-related and patient-reported outcomes.

This study has several strengths and limitations. Strengths include its multicenter prospective design, the standardized delivery of the NEP, and the stratified analysis according to baseline dialysis adequacy, which allowed a physiologically grounded interpretation of heterogeneous intervention effects.

Among the limitations, allocation to intervention or control groups was determined by center rather than individual randomization, which may limit causal inference. In addition, because the intervention was delivered at the center level, differences in local clinical routines, staff expertise, and the way standard care was provided across centers may have influenced outcomes independently of the NEP. Although key dialysis prescription parameters were standardized across centers, residual center-related confounding cannot be excluded. Although an *a priori* sample size calculation was performed for the overall comparison between groups, the Kt/V-stratified subgroup analyses were exploratory and were not specifically powered. Accordingly, findings derived from these subgroup analyses, particularly those from strata with smaller sample sizes, should be interpreted cautiously. On the other hand, improvements in dialysis adequacy were not consistently maintained over time, likely reflecting attenuation of educational effects in the absence of sustained reinforcement and a potential Hawthorne effect (66–68). The lack of direct body composition assessment limits mechanistic interpretation of changes in urea distribution volume and Kt/V (35, 36, 69). Furthermore, the inclusion of clinically stable patients from a specific geographic area may limit generalizability, and residual confounding related to the complex interplay between nutritional status, inflammation, anemia, and dialysis adequacy cannot be fully excluded (22, 28, 32, 37). In addition, because participants in the intervention group were aware that the study focused on nutrition education delivered by an external researcher and following the site's protocol, social desirability bias cannot be excluded. Therefore, improvements in dietary variables should be interpreted as self-reported intake rather than direct objective confirmation of actual dietary behavior. Finally, because no repeated-measures ANOVA or mixed-effects model was applied, any interpretation suggesting temporal trends should be interpreted cautiously. The results regarding Q1 group should also be interpreted with caution, given its small sample size.

## 5 Conclusions

In conclusion, structured nutrition education may contribute to improvements in dietary quality, metabolic control, and dialysis-related outcomes in patients undergoing maintenance HD, with effects that vary according to baseline dialysis adequacy. The main contribution of this work is to show that baseline Kt/V may help identify different patterns of response to the same educational intervention, which supports a more individualized approach to nutritional counseling in routine HD care. However, because of the center-based non-randomized design, the exploratory nature and limited power of the Kt/V-stratified subgroup analyses, the limited sample size of some Kt/V strata, and the lack of direct fluid-status or body-composition measurements, these effects should be interpreted cautiously.

## 6 Practical application

From a clinical perspective, nutrition education should be considered an ongoing component of routine HD care rather than a uniform, one-size-fits-all intervention. The findings of this study suggest that nutritional counseling may need to be prioritized and adapted according to patients' baseline clinical status, with emphasis on dietary quality and nutrient selection in more vulnerable patients and on fluid and treatment-related behaviors in clinically stable individuals. Integrating structured nutrition education into standard dialysis workflows may help reinforce dietary adherence and support comprehensive patient management beyond dialysis prescription alone.

## Data Availability

The raw data supporting the conclusions of this article will be made available by the authors, without undue reservation.

## References

[1] Kovesdy CP. Epidemiology of chronic kidney disease: an update 2022. Kidney Int Suppl. (2022) 12:7–11. doi: 10.1016/j.kisu.2021.11.003 35529086 PMC9073222

[2] Guo J Liu Z Wang P Wu H Fan K Jin J . Global, regional, and national burden inequality of chronic kidney disease, 1990–2021: a systematic analysis for the global burden of disease study 2021. Front Med. (2025) 11:1501175. doi: 10.3389/fmed.2024.1501175 PMC11774877 39882527

[3] Francis A Harhay MN Ong ACM Tummalapalli SL Ortiz A Fogo AB . Chronic kidney disease and the global public health agenda: an international consensus. Nat Rev Nephrol. (2024) 20:473–85. doi: 10.1038/s41581-024-00820-6 38570631

[4] Chandra P Sachan N Saraswat N Vyawahare N. A detailed review of molecular pathways and mechanisms responsible for the development and aggravation of neuropathy and nephropathy in diabetes. Curr Mol Pharmacol. (2024) 17:e280323215026. doi: 10.2174/1874467217666230328084215 37018534

[5] Ikizler TA Burrowes JD Byham-Gray LD Campbell KL Carrero J-J Chan W . KDOQI clinical practice guideline for nutrition in CKD: 2020 Update. Am J Kidney Dis. (2020) 76:S1–S107. doi: 10.1053/j.ajkd.2020.05.006 32829751

[6] Roumeliotis S Divani M Stamellou E Liakopoulos V. Genomics in diabetic kidney disease: a 2024 update. Curr Genomics. (2024) 25:153–7. doi: 10.2174/0113892029300247240325080421 39086997 PMC11288163

[7] Dwivedi J Kaushal S Wal P Singh DP Gupta P Sowjanya P . Tailored therapies for hereditary diabetes: unraveling the genetic underpinnings of MODY and neonatal diabetes. Curr Gene Ther. (2025) 25. doi: 10.2174/0115665232381943250825055516 40910293

[8] Elhassan S Abdelhadi IAA Mohamed NNS Mohammed AMA Mohammed WAO Abdalla HHM . Malnutrition among patients with end-stage renal disease in war 2024: the role of healthcare access, dialysis, gender, and economic disparities. Int J Equity Health. (2025) 24:298. doi: 10.1186/s12939-025-02680-3 41168820 PMC12577354

[9] Arroyo-Serrano P Alonso-Dominguez R Mas-Fontao S Gonzalez-Parra E Sánchez-Tocino ML. Nutritional strategies to address malnutrition in dialyses patients: a systematic review. Nutrients. (2025) 17:3478. doi: 10.3390/nu17213478 41228549 PMC12608553

[10] Sarav M Kovesdy CP. Protein Energy Wasting in Hemodialysis Patients. Clin J Am Soc Nephrol. (2018) 13:1558–60. doi: 10.2215/CJN.02150218 29954825 PMC6218831

[11] Disthabanchong S Vantanasiri K Khunapornphairote S Chansomboon P Buachum N Saeseow S. Severe hyperparathyroidism is associated with nutritional impairment in maintenance hemodialysis patients. Front Nutr. (2022) 9:933918. doi: 10.3389/fnut.2022.933918 36176632 PMC9513451

[12] Pham Thi Lan A Truong Thanh A Luong Ngoc Q Pham Nhat T Doan Duy T. Prevalence and factors associated with malnutrition among hemodialysis patients in a single hemodialysis center in Vietnam: a cross-sectional study. Medicine. (2024) 103:e37679. doi: 10.1097/MD.0000000000037679 38579083 PMC10994475

[13] Hamdan Z Nazzal Z Al-Amouri FM Ishtayah S Sammoudi S Bsharat L . Factors associated with malnutrition inflammation score among hemodialysis patients: A cross-sectional investigation in tertiary care hospital, Palestine. PLoS ONE. (2025) 20:e0317132. doi: 10.1371/journal.pone.0317132 39854402 PMC11761151

[14] Picard K Mager D Richard C. How food processing impacts hyperkalemia and hyperphosphatemia management in chronic kidney disease. Can J Diet Pract Res. (2020) 81:132–6. doi: 10.3148/cjdpr-2020-003 32072822

[15] Gotch FA Sargent JA. A mechanistic analysis of the National Cooperative Dialysis Study (NCDS). Kidney Int. (1985) 28:526–34. doi: 10.1038/ki.1985.160 3934452

[16] Zhu C Xue C Lan N Zeng F Wang H Yang B. Dialysis adequacy revisited: Kt/V's blind spot for phosphorus and iodine. J Transl Intern Med. (2025) 13:394–396. doi: 10.1515/jtim-2025-0050 PMC12569571 41169520

[17] Hoshino J. Renal rehabilitation: exercise intervention and nutritional support in dialysis patients. Nutrients. (2021) 13:1444. doi: 10.3390/nu13051444 33923264 PMC8145577

[18] Chen ZQ Luo L Chen XX Zhang XY Yin SQ Xiao GH . Dietary nutrient intake and nutritional status in maintenance hemodialysis patients: a multicenter cross-sectional survey. Ren Fail. (2024) 46:2363589. doi: 10.1080/0886022X.2024.2363589 38874093 PMC11182067

[19] Ouirdani M Boutib A Azizi A Chergaoui S Saad EM Hilali A . Impact of nutrition education on various health-related components of hemodialysis patients: a systematic review. Healthc Basel Switz. (2024) 12:1197. doi: 10.3390/healthcare12121197 PMC11203892 38921311

[20] Sultan B Froelicher ES. Effectiveness of patient education on adherence to treatment regimen and quality of life in hemodialysis patients: a systematic review and meta-analysis. Minerva Urol Nephrol. (2025) 77:762–75. doi: 10.23736/S2724-6051.24.05718-5 40052258

[21] Sajjadi SL Ghafourifard M Tayebi Khosroshahi H. A randomized controlled clinical trial of individualized patient education on hemodialysis adequacy and interdialytic weight gain. J Caring Sci. (2025) 14:5–13. doi: 10.34172/jcs.025.33604 40391310 PMC12085766

[22] Padial M Avesani CM García-Testal A Cana-Poyatos A Lindholm B Segura-Ortí E. Dietary needs, barriers, and facilitators among patients on hemodialysis and their caregivers: the GoodRENal Project in Spain. J Ren Nutr. (2025) 35:337–43. doi: 10.1053/j.jrn.2024.08.005 39237029

[23] Hunter EG Shukla A Andrade JM. Barriers to and strategies for dietary adherence: a qualitative study among hemodialysis/peritoneal dialysis patients and health care providers. J Ren Nutr. (2023) 33:682–90. doi: 10.1053/j.jrn.2023.06.007 37315706

[24] Lambert K Bahceci S Harrison H Chan M Scholes-Robertson N Johnson DW . Commentary on the 2020 update of the KDOQI clinical practice guideline for nutrition in chronic kidney disease. Nephrol Carlton Vic. (2022) 27:537–40. doi: 10.1111/nep.14025 PMC9303594 35118773

[25] Charkviani M Thongprayoon C Tangpanithandee S Krisanapan P Miao J Mao MA . Effects of Mediterranean diet, DASH diet, and plant-based diet on outcomes among end stage kidney disease patients: a systematic review and meta-analysis. Clin Pract. (2022) 13:41–51. doi: 10.3390/clinpract13010004 36648844 PMC9844348

[26] Garagarza C Valente A Caetano C Ramos I Sebastião J Pinto M . Mediterranean Diet: A Dietary Pattern Related to Nutritional Benefits for Hemodialysis Patients. J Ren Nutr.. (2023) 33:472–81. doi: 10.1053/j.jrn.2023.01.006 36731683

[27] Akhtar T Froelicher ES Victor G. Effectiveness of Nutritional Education for Hemodialysis Patients with Chronic Kidney Disease: A Systematic Review and Meta-Analysis: Nutritional Education in Haemodialysis: A Review and Meta-Analysis. J Health Rehabil Res. (2024) 4:1–8. doi: 10.61919/jhrr.v4i3.1324

[28] Piccoli GB Moio MR Fois A Sofronie A Gendrot L Cabiddu G . The diet and haemodialysis dyad: three eras, four open questions and four paradoxes. A narrative review, towards a personalized, patient-centered approach. Nutrients. (2017) 9:372. doi: 10.3390/nu9040372 28394304 PMC5409711

[29] Walser M. Urea metabolism in chronic renal failure. J Clin Invest. (1974) 53:1385–92. doi: 10.1172/JCI107687 4825230 PMC302627

[30] de Luis D Bustamante J. [Nutritional aspects in renal failure]. Nefrol Publicacion Of Soc Espanola Nefrol. (2008) 28:333–42. doi: 10.1007/BF03255892 18590502

[31] Teixeira Nunes F de Campos G Xavier de. Paula SM Merhi VAL Portero-McLellan KC da Motta DG . Dialysis adequacy and nutritional status of hemodialysis patients Hemodial. Int Int Symp Home Hemodial. (2008) 12:45–51. doi: 10.1111/j.1542-4758.2008.00239.x 18271840

[32] Oriol-Vila E Rota-Musoll L Molina-Robles E Roure-Pujol C Chiverches-Pérez E. Educational interventions for haemodialysis patients in the transplant process: a systematic review. Nurs Open. (2024) 11:e70104. doi: 10.1002/nop2.70104 39665262 PMC11635394

[33] El Chamieh C Liabeuf S Massy Z. Uremic toxins and cardiovascular risk in chronic kidney disease: what have we learned recently beyond the past findings? Toxins. (2022) 14:280. doi: 10.3390/toxins14040280 35448889 PMC9028122

[34] Visiedo L López F Rivas-Ruiz F Tortajada B Giménez R Abilés J . Efecto de un programa de intervención nutricional personalizado en el estado nutricional, calidad de vida y mortalidad en pacientes en hemodiálisis. Nutr Hosp. (2023) 40:1229–35. doi: 10.20960/nh.04756 37705451

[35] Daugirdas JT Greene T Depner TA Levin NW Chertow GM. Modeled urea distribution volume and mortality in the HEMO study. Clin J Am Soc Nephrol CJASN. (2011) 6:1129–38. doi: 10.2215/CJN.06340710 21511841 PMC3087780

[36] Hecking M Karaboyas A Antlanger M Saran R Wizemann V Chazot C . Significance of interdialytic weight gain versus chronic volume overload: consensus opinion. Am J Nephrol. (2013) 38:78–90. doi: 10.1159/000353104 23838386

[37] Bossola M Pepe G Antocicco M Severino A Di Stasio E. Interdialytic weight gain and educational/cognitive, counseling/behavioral and psychological/affective interventions in patients on chronic hemodialysis: a systematic review and meta-analysis. J Nephrol. (2022) 35:1973–83. doi: 10.1007/s40620-022-01450-6 36112313 PMC9584995

[38] Wizemann V Wabel P Chamney P Zaluska W Moissl U Rode C . The mortality risk of overhydration in haemodialysis patients. Nephrol Dial Transplant. (2009) 24:1574–9. doi: 10.1093/ndt/gfn707 19131355 PMC2668965

[39] Zoccali C Tripepi G Carioni P Mallamaci F Savoia M. Usvyat LS. Fluid overload trajectories and mortality in hemodialysis patients. J Intern Med. (2025) 297:201–12. doi: 10.1111/joim.20049 39732505

[40] Flythe JE Kimmel SE Brunelli SM. Rapid fluid removal during dialysis is associated with cardiovascular morbidity and mortality. Kidney Int. (2011) 79:250–7. doi: 10.15215/aupress/9781897425909.041 20927040 PMC3091945

[41] Barzegar H Moosazadeh M Jafari H Esmaeili R. Evaluation of dialysis adequacy in hemodialysis patients: a systematic review. Urol J. (2016) 13:2744–9. 27576879

[42] Pérez-Torres A Caverni-Muñoz A González García E. Mediterranean diet and chronic kidney disease (CKD): a practical approach. Nutrients. (2022) 15:97. doi: 10.3917/prat.097.0042 36615755 PMC9824533

[43] López-Gil JF García-Hermoso A Martínez-González MÁ Rodríguez-Artalejo F. Mediterranean diet and cardiometabolic biomarkers in children and adolescents: a systematic review and meta-analysis. JAMA Netw Open. (2024) 7:e2421976. doi: 10.1001/jamanetworkopen.2024.21976 38995643 PMC11245727

[44] Snetselaar LG de Jesus JM DeSilva DM Stoody EE. Dietary guidelines for Americans, 2020–2025. Nutr Today. (2021) 56:287–95. doi: 10.1097/NT.0000000000000512 34987271 PMC8713704

[45] Mazzocchi A Leone L Agostoni C Pali-Schöll I. The secrets of the Mediterranean Diet. Does [only] olive oil matter? Nutrients. (2019) 11:2941. doi: 10.3390/nu11122941 31817038 PMC6949890

[46] Kalantar-Zadeh K Kopple JD Block G Humphreys MH. A malnutrition-inflammation score is correlated with morbidity and mortality in maintenance hemodialysis patients. Am J Kidney Dis. (2001) 38:1251–63. doi: 10.1053/ajkd.2001.29222 11728958

[47] Ussia S Ritorto G Mollace R Serra M Tavernese A Altomare C . Exploring the benefits of extra virgin olive oil on cardiovascular health enhancement and disease prevention: a systematic review. Nutrients. (2025) 17:1843. doi: 10.3390/nu17111843 40507112 PMC12158199

[48] Kris-Etherton PM Harris WS Appel LJ. Omega-3 fatty acids and cardiovascular disease. Arterioscler Thromb Vasc Biol. (2003) 23:151–2. doi: 10.1161/01.ATV.0000057393.97337.AE 12588750

[49] Bucharles SGE Wallbach KKS Moraes TP de Pecoits-Filho R. Hypertension in patients on dialysis: diagnosis, mechanisms, and management. J Bras Nefrol. (2019) 41:400–11. doi: 10.1590/2175-8239-jbn-2018-0155 30421784 PMC6788847

[50] Fotiadou E Georgianos PI Vaios V Sgouropoulou V Divanis D Karligkiotis A . Feeding during dialysis increases intradialytic blood pressure variability and reduces dialysis adequacy. Nutrients. (2022) 14:1357. doi: 10.3390/nu14071357 35405970 PMC9002965

[51] Obi Y Qader H Kovesdy CP Kalantar-Zadeh K. Latest consensus and update on protein-energy wasting in chronic kidney disease. Curr Opin Clin Nutr Metab Care. (2015) 18:254–62. doi: 10.1097/MCO.0000000000000171 25807354 PMC4506466

[52] Carrero JJ Stenvinkel P Cuppari L Ikizler TA Kalantar-Zadeh K Kaysen G . Etiology of the protein-energy wasting syndrome in chronic kidney disease: a consensus statement from the International Society of Renal Nutrition and Metabolism (ISRNM). J Ren Nutr. (2013) 23:77–90. doi: 10.1053/j.jrn.2013.01.001 23428357

[53] Mushtaq M Usmani MR Hameed N Anwar A Hashmi AA. Serum vitamin b12 deficiency in chronic hemodialysis patients. Cureus. (2024) 16:e58751. doi: 10.7759/cureus.58751 38779272 PMC11110947

[54] Auerbach M Ballard H Glaspy J. Clinical update: intravenous iron for Anaemia. Lancet. (2007) 369:1502–4. doi: 10.1016/S0140-6736(07)60689-8 17482969

[55] Satirapoj B Apiyangkool T Thimachai P Nata N Supasyndh O. Intradialytic oral nutrition effects on malnourished hemodialysis patients: a randomized trial. Sci Rep. (2024) 14:21400. doi: 10.1038/s41598-024-72402-2 39271736 PMC11399429

[56] Hanna RM Streja E Kalantar-Zadeh K. Burden of anemia in chronic kidney disease: beyond erythropoietin. Adv Ther. (2021) 38:52–75. doi: 10.1097/01.NUMA.0000752808.43608.03 33123967 PMC7854472

[57] Sahathevan S Khor B-H Ng H-M Abdul Gafor AH Mat Daud ZA Mafra D . Understanding development of malnutrition in hemodialysis patients: a narrative review. Nutrients. (2020) 12:3147. doi: 10.3390/nu12103147 33076282 PMC7602515

[58] Biruete A Hill Gallant KM Lloyd L Meade A Moe SM St-Jules DE . ‘Phos'tering a clear message: the evolution of dietary phosphorus management in chronic kidney disease. J Ren Nutr. (2023) 33:S13–20. doi: 10.1053/j.jrn.2023.05.004 37343779 PMC10728341

[59] Kalantar-Zadeh K. Patient education for phosphorus management in chronic kidney disease. Patient Prefer Adherence. (2013) 7:379–90. doi: 10.2147/PPA.S43486 23667310 PMC3650565

[60] Lim E Hyun S Lee JM Kim S Lee M-J Lee S-M . Effects of education on low-phosphate diet and phosphate binder intake to control serum phosphate among maintenance hemodialysis patients: a randomized controlled trial. Kidney Res Clin Pract. (2018) 37:69–76. doi: 10.23876/j.krcp.2018.37.1.69 29629279 PMC5875578

[61] Joshi U Subedi R Poudel P Ghimire PR Panta S Sigdel MR. Assessment of quality of life in patients undergoing hemodialysis using WHOQOL-BREF questionnaire: a multicenter study. Int J Nephrol Renov Dis. (2017) 10:195–203. doi: 10.2147/IJNRD.S136522 28790861 PMC5529382

[62] Testa MA Simonson DC. Assessment of quality-of-life outcomes. N Engl J Med. (1996) 334:835–40. 8596551 10.1056/NEJM199603283341306

[63] Yonata A Islamy N Taruna A Pura L. Factors affecting quality of life in hemodialysis patients. Int J Gen Med. (2022) 15:7173–8. doi: 10.2147/IJGM.S375994 36118180 PMC9480587

[64] Jung H-Y Jeon Y Park Y Kim YS Kang S-W Yang CW . Better quality of life of peritoneal dialysis compared to hemodialysis over a two-year period after dialysis initiation. Sci Rep. (2019) 9:10266. doi: 10.1038/s41598-019-46744-1 31312004 PMC6635359

[65] Bandura A. Self-efficacy: The exercise of control. New York, NY: W H Freeman/Times Books/ Henry Holt & Co (1997). 604 p.

[66] Ren Q Shi S Yan C Liu Y Han W Lin M . Self-management micro-video health education program for hemodialysis patients. Clin Nurs Res. (2022) 31:1148–57. doi: 10.1177/10547738211033922 34282644

[67] McCambridge J Witton J Elbourne DR. Systematic review of the Hawthorne effect: new concepts are needed to study research participation effects. J Clin Epidemiol. (2014) 67:267–77. doi: 10.1016/j.jclinepi.2013.08.015 24275499 PMC3969247

[68] Kelly JT Conley M Hoffmann T Craig JC Tong A Reidlinger DP . A Coaching Program to Improve Dietary Intake of Patients with CKD: ENTICE-CKD. Clin J Am Soc Nephrol. (2020) 15:330–40. doi: 10.2215/CJN.12341019 32111701 PMC7057309

[69] Pérez-García R Jaldo M Alcázar R de Sequera P Albalate M Puerta M . Unlike Kt, high Kt/V is associated with greater mortality: the importance of low V. Nefrologia. (2019) 39:58–66. doi: 10.1016/j.nefroe.2018.04.011 30075965

