# Correction: Effects of a structured nutrition education program on dialysis adequacy and clinical outcomes in maintenance hemodialysis patients

**DOI:** 10.3389/fnut.2026.1937041

**Published:** 2026-07-24

**Authors:** Desirée Victoria-Montesinos, Pura Ballester, Valentín Cadenas-García, Juana M. Morillas-Ruiz

**Affiliations:** 1 Universidad Católica San Antonio de Murcia Facultad de Ciencias de la Salud, Murcia, Spain; 2 Servicio de Análisis Clínicos, Hospital Rafael Méndez de Lorca, Área de Salud III, Servicio Murciano de Salud, Murcia, Spain

**Keywords:** dialysis adequacy, Kt/v, maintenance hemodialysis, nutrition education, renal physiology

Affiliation “Facultad de Deportes, Universidad Católica San Antonio de Murcia, Murcia, Spain” was erroneously included in the paper. This affiliation has now been removed for author Juana M. Morillas-Ruiz. The author Juana M. Morillas-Ruiz should be affiliated with affiliation 1 “Universidad Católica San Antonio de Murcia Facultad de Ciencias de la Salud, Murcia, Spain.”

The original version of this article has been updated.

